# Confounders in the assessment of the renal effects associated with low-level urinary cadmium: an analysis in industrial workers

**DOI:** 10.1186/1476-069X-10-37

**Published:** 2011-05-14

**Authors:** Nahida Haddam, Sekkal Samira, Xavier Dumont, Abdesselem Taleb, Dominique Lison, Vincent Haufroid, Alfred Bernard

**Affiliations:** 1 Laboratory of Medical Toxicology, Faculty of Medicine, University Abou Bekr Belkaid, Tlemcen, Algeria; 2 Institute of Biology, Faculty of Science, University Abou Bekr Belkaid, Tlemcen, Algeria; 3 Unit of Toxicology and Applied Pharmacology, Catholic University of Louvain, Brussels, Belgium

## Abstract

**Background:**

Associations of proteinuria with low-level urinary cadmium (Cd) are currently interpreted as the sign of renal dysfunction induced by Cd. Few studies have considered the possibility that these associations might be non causal and arise from confounding by factors influencing the renal excretion of Cd and proteins.

**Methods:**

We examined 184 healthy male workers (mean age, 39.5 years) from a zinc smelter (n = 132) or a blanket factory (n = 52). We measured the concentrations of Cd in blood (B-Cd) and the urinary excretion of Cd (U-Cd), retinol-binding protein (RBP), protein HC and albumin. Associations between biomarkers of metal exposure and urinary proteins were assessed by simple and multiple regression analyses.

**Results:**

The medians (interquartile range) of B-Cd (μg/l) and U-Cd (μg/g creatinine) were 0.80 (0.45-1.16) and 0.70 (0.40-1.3) in smelter workers and 0.66 (0.47-0.87) and 0.55 (0.40-0.90) in blanket factory workers, respectively. Occupation had no influence on these values, which varied mainly with smoking habits. In univariate analysis, concentrations of RBP and protein HC in urine were significantly correlated with both U-Cd and B-Cd but these associations were substantially weakened by the adjustment for current smoking and the residual influence of diuresis after correction for urinary creatinine. Albumin in urine did not correlate with B-Cd but was consistently associated with U-Cd through a relationship, which was unaffected by smoking or diuresis. Further analyses showed that RBP and albumin in urine mutually distort their associations with U-Cd and that the relationship between RBP and Cd in urine was almost the replicate of that linking RBP to albumin

**Conclusions:**

Associations between proteinuria and low-level urinary Cd should be interpreted with caution as they appear to be largely driven by diuresis, current smoking and probably also the co-excretion of Cd with plasma proteins.

## Introduction

Cadmium (Cd) is a widespread pollutant that accumulates in the soil, the food chain and ultimately in the human body. Other major sources of exposure to Cd are cigarette smoking and the use or production of Cd in various industrial settings. Cd is a highly toxic metal which at high doses can cause damage on virtually all organs and biological systems. At current human exposure levels in the industry or environment, it is assumed that Cd primarily damages the kidneys and especially the proximal tubular cells where the metal selectively concentrates. The hallmark of Cd nephrotoxicity is an increased urinary excretion of low-molecular-weight (LMW) proteins such as β_2_-microglobulin, retinol-binding protein (RBP) or protein HC. This increased excretion of LMW proteins in urine referred to as "tubular proteinuria" results from a defective protein reabsorption by the damaged tubular cells [1-3].

Studies among industrial workers and populations living in heavily polluted areas have clearly shown that Cd causes tubular dysfunction in a dose-dependent manner, the tubular proteinuria developing only when the renal accumulation has reached a critical threshold. In adult workers exposed mainly by inhalation, the critical concentration of Cd in renal cortex from which tubular proteinuria is likely to develop has been estimated at approximately 200 ppm (μg/g wet weight of renal cortex) [4]. The corresponding threshold level for urinary Cd, an indicator of the Cd body burden in industrial workers, has been estimated at 10 μg/creatinine [5,6]. This U-Cd threshold level has been confirmed in a recent study, which estimated the U-Cd benchmark dose for the LMW proteinuria induced by Cd at 12-13 μg/g creatinine with a lower confidence interval limit of about 5-6 μg/g creatinine [7].

While surveys among Cd workers have derived rather consistent estimates of U-Cd threshold for the development of LMW proteinuria, studies among the general population have with time reported increasingly lower U-Cd threshold values despite the use of the same renal biomarkers [8-10]. Some studies have even derived U-Cd threshold levels within the range of values now prevailing in industrialized countries [11,12], which suggests that a substantial fraction of the human population might be at risk of Cd nephrotoxicity. Recent studies even suggest that the cumulative exposure of the general population to Cd might increase the risks of chronic renal diseases [13,14].

A possibility, however, which has received little attention is that associations between urinary Cd and renal markers at very low exposure levels might not be causal but simply reflect the elimination of Cd by the kidneys and in particular the co-excretion of the metal with urinary proteins. Because most Cd circulating in plasma is bound to proteins and especially to the LMW metallothionein, a variation in the tubular reabsorption of these LMW proteins in health or disease should unavoidably result in an increased co-excretion of Cd and LMW proteins in urine [15]. There is indeed no reason to believe that the renal handling of proteins binding Cd would be different from that of other plasma proteins apart some differences related to the size and charge, which are the main factors governing the renal handling of proteins. Other important confounders which also need to be carefully evaluated in causal inference are the diuresis and chronic smoking, which is a contributor to Cd exposure but also to renal dysfunction [7,16-19].

The objective of the current study focused on workers with low exposure to Cd was to further explore the factors that might confound the associations between Cd and proteins in urine. Screening for tubular proteinuria was based on the measurement of RBP and protein HC in urine, two reliable biomarkers for screening tubular dysfunction induced by Cd. Different models were built to detect the confounding effects which might arise from current or cumulative smoking and from an insufficient adjustment for diuresis. We also measured urinary albumin to identify possible co-excretion mechanisms related to changes in the filtered load and tubular uptake of plasma proteins.

## Materials and methods

### Study population

The studied population included 134 healthy male workers, who had been working in the zinc smelter of Ghazaouet in Algeria. In 2009 when the workers were examined, the smelter annual production was of 36.000 tons of zinc and 72.000 tons of sulphuric acid. The production of Cd ceased in 1999 and about half of the studied workers were not yet employed in the plant at that time. The reference group was constituted of 52 male workers from an acrylic blanket factory located in Tlemcen, a city 60 km from Ghazaouet. Biological data and information about the duration of employment and the smoking history were obtained in the framework of the workers' health surveillance programme that was implemented in each factory in order to comply with the regulation. These data were sent anonymously by the factory heath care units.

### Protocol

Samples of urine (untimed samples) and blood for metal and protein analyses were collected in the framework of the periodic medical surveillance of workers. Cd was measured in urine by means of inductively coupled argon plasma mass spectrometry (ICP-MS) with an Agilent 7500 instrument. Briefly, urine specimens (500 μl) were diluted quantitatively 1+9 with a HNO_3 _1%, HCl 0.5% solution containing Sc, Ge, Rh and Ir as internal standards. The detection and quantification limits were 0.02 and 0.05 μg/l, respectively. The laboratory has performed successfully in external quality assessment programmes of the Institute for Occupational, Environmental and Social Medicine of the University of Erlangen, Germany (G-EQUAS programme) and in the PCI and QMEQUAS programmes of the "Institut National de Santé Publique", Québec. Creatinine was determined by a modified Jaffé reaction using a Beckman Synchron LX 20 analyser (Beckman Coulter GmbH, Krefeld, Germany). This method has an excellent formulation, minimizing interference by protein, bilirubin and glucose. We measured the concentrations of RBP by latex immunoassay with a detection and quantification limits of 0.5 and 2.5 μg/l respectively, based on a five-fold dilution of urine.

### Statistics

Age, body mass index (BMI), duration of exposure and number of pack-years were described as mean with standard deviation. Concentrations of biomarkers in urine and blood were described as median with interquartile range (IQR). Associations between biomarkers and their potential predictors were assessed by Pearson's correlation analysis. We used two-way ANOVA to evaluate changes in biomarkers associated with working in smelter, smoking status (never-, ex- and current smoker) and the possible interactions between these two factors. Comparison between groups of smokers was performed using the Scheffé's multiple comparison test for continuous variables and by the chi-square test for dichotomic variables. Associations between concentrations of proteins and U-Cd were assessed in stepwise regression analyses by testing as potential predictors age, BMI, U-Cd, B-Pb, U-Zn, current smoking, and the number of pack-years, which were introduced in the models as dummy variables (pack-years > 1-15 and > 15). We built three models in order to assess the influence of diuresis on the concentrations of biomarkers expressed per liter or per g of creatinine. In model 1, analyses were performed by expressing the concentrations of urinary biomarkers per liter or per g of creatinine without any further adjustment for the variable dilution of urine. In the model 2, the urinary creatinine was added to the list of potential predictors to adjust for the influence (concentration per liter) or the residual influence (concentration per g of creatinine) of diuresis on the concentrations of urinary proteins. In the model 3, U-Cd expressed per liter or g of creatinine was tested after a preliminary adjustment for urinary creatinine on the basis of the simple regression coefficient. The same analyses were performed with B-Cd at the exception that only models adjusting the concentrations of proteins in urine were used (models 1 and 2). Independent variables in multiple regression analyses were entered at a P value of 0.25 and kept in the model at P value < 0.05. With the exception of age and urinary RBP which were normally distributed, all variables were normalized by log transformation. The level of statistical significance was set at p < 0.05. Statistical analyses were performed by using SAS version 9.1.3 (SAS International, Cary, NC).

## Results

**Figure 1:**
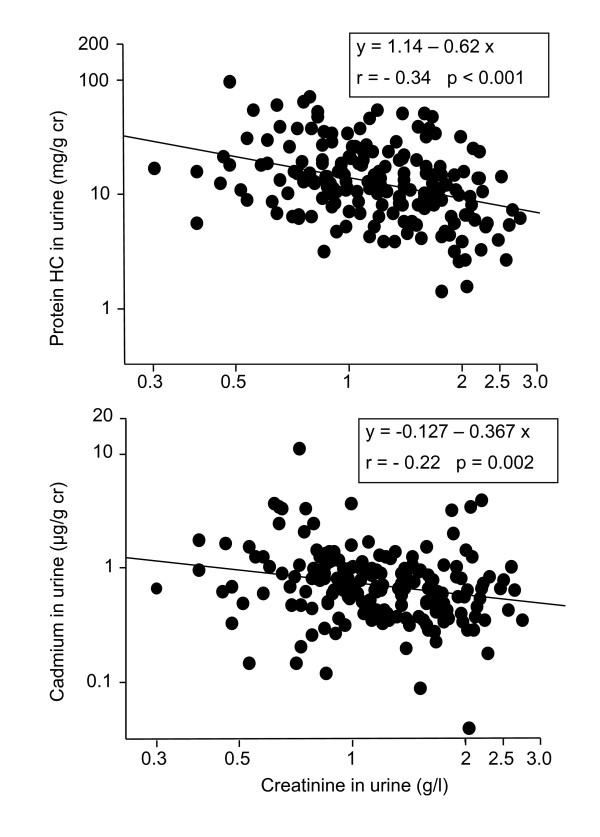
**Associations of the creatinine-adjusted concentrations of protein HC and Cd in urine with the urinary concentration of creatinine**.

Table 1 compares the characteristics and biomarker levels between the smelter and blanket factory workers stratified according to their smoking status. At the exception of the blood lead level, which was higher in smelter workers, there were no significant differences between the two groups of workers regarding the metal exposure and renal biomarkers. The only noticeable differences concerned current smokers who had significantly higher levels of Cd in blood and urine and excreted also more RBP and protein HC in urine. The univariate analyses detailed in Table 2 show that urinary RBP, protein HC and albumin correlate significantly with urinary Cd. Urinary protein HC was also positively associated with age and pack-years, two factors which had no influence on urinary RBP and albumin. Protein HC and RBP in urine were also associated with Cd in blood while urinary RBP was associated with lead in blood. Of note also, the concentrations of Cd and protein HC in urine, though expressed per gram of creatinine, showed negative associations with urinary creatinine. These associations, illustrated in Figure 1, mean that expressing these two analytes per g of creatinine results in an over-adjustment that does not completely eliminate the influence of diuresis. The association between Cd and creatinine in urine, which was positive with U-Cd expressed per liter (coefficient, 0.60) turned to be negative after adjustment for urinary creatinine (Figure 1). A similar phenomenon was observed with urinary protein HC which surprisingly was already negatively correlated with urinary creatinine when expressed per liter (coefficient, -0.44).

**Table 1:** Characteristics of workers and their biological parameters

	Acrylic blanket factory workers			Zinc smelter workers			Two-way ANOVA		
	**Never-smokers**	**Ex-smokers**	**Current smokers**	**Never-smokers**	**Ex-smokers**	**Current smokers**	**Occupation**	**Smoking**	**Interaction**

N (%)	19 (36.5)	12 (23.1)	21 (40.4)	41 (30.6)	38 (28.4)	55 (41.0)			

Age (years)	40.6 (10.1)	42.4 (8.4)	39.6 (8.4)	35.5 (10.1)	42.7 (10.6)	39.0 (10.9)	0.29	0.10	0.36

BMI (kg/m²)	25.1 (3.7)	25.2 (4.0)	23.3 (3.4)*	26.1 (2.9)	25.8 (3.1)	24.3 (3.3)*	0.07	0.004	0.94

Employment (years)	10.6 (7.3)	12 (8.3)	9.6 (7.1)	9.4 (10.3)	14.7 (8.8)	12.2 (9.3)	0.36	0.22	0.46

Pack-years (N)	-	14.9 (9.4)	18.8 (11.8)	-	14.2 (7.6)	17.5 (9.20)	0.87	-	-

B-Pb (μg/l)	46 (36-58)	55 (44-58)	49 (36-59)	95 (76-137)	89(70-116)	116 (86-143)	<0.001	0.51	0.34

B-Cd (μg/l)	0.40 (0.30-0.45)	0.50 (0.45-0.85)	0.90 (0.70-1.23)* ^$^	0.50 (0.30-0.70)	0.60 (0.40-0.80)	1.20 (0.80-1.70)* ^$^	0.09	<0.001	0.92

U-Cd (μg/g cr)	0.53 (0.37-0.68)	0.78 (0.67-0.90)	0.65 (0.48-1.02)	0.48 (0.37-0.81)	0.76 (0.55-1.18)*	1.00 (0.70-1.37)*	0.37	<0.001	0.19

U-Zn (mg/g cr)	0.23 (0.18-0.35)	0.28 (0.27-0.39)	0.26 (0.21-0.32)	0.26 (0.16-0.40)	0.28 (0.21-0.48)	0.32 (0.20-0.47)	0.34	0.19	0.38

U-ALB (mg/g cr)	5.4 (3.4-8.5)	6.9 (4.7-9.1)	5.6 (4.0-9.6)	7.9 (5.0-10.8)	8.3 (4.2-12.4)	7.8 (4.7-14.3)	0.09	0.28	0.97

U-Protein-HC (mg/g cr)	9.3 (5.9-17.4)	15.5 (10.9-18.6)	13.1 (8.6-22.7)	9.4 (5.5-14.1)	12.6 (8.5-20.8)	16.0 (9.9-27.5)*	0.82	0.01	0.63

U-RBP (mg/g cr)	120 (91.3-146)	149 (107-232)	153 (102-194)	122 (85-161)	123 (90-152)	172 (141-200)	0.74	0.01	0.99

U-Creatinine(g/l)	1.14 (0.91-1.73)	1.10 (0.94-1.33)	1.18 (0.95-1.51)	1.31 (0.88-1.78)	1.27 (0.85-1.66)	1.08 (0.78-1.51)	0.73	0.44	0.77

* significantly different from neversmokers $significantly different from ex-smokers by the test of Tukey-Kramer. Age, body mass index (BMI), duration of exposure and number of pack-years are given as mean with standard deviation. Concentrations of biomarkers in urine and blood are described as median with interquartile range (IQR).

**Table 2:** Pearson's correlation coefficients between urinary proteins and their potential predictors

	Age	Log BMI	Pack-years	Log B-Pb	Log B-Cd	Log U-Cr	Log U-Cd	Log U-Zn	Log U-RBP	Log U-Protein-HC
Log BMI	0.13									
Pack-years	0.27***	-0.15*								
Log B-Pb	-0.05	0.03	-0.02							
Log B-Cd	-0.08	-0.19*	0.42***	0.25***						
Log U-Cr	-0.22**	0.14	-0.10	-0.03	-0.004					
Log U-Cd	0.24**	-0.18*	0.34***	0.17*	0.43***	- 0.22**				
Log U-Zn	-0.02	-0.08	0.08	0.09	0.11	- 0.05	0.16*			
Log U-RBP	0.05	0.08	0.14	0.17*	0.26***	-0.14	0.21**	0.03		
Log U-Protein-HC	0.23***	0.02	0.23**	-0.01	0.22**	-0.34***	0.23**	0.06	0.45***	
Log U-ALB	0.03	-0.004	0.11	0.04	0.14	-0.005	0.15*	-0.06	0.29***	0.31***

For the units of parameters, see Table 1. *p < 0.05, **p < 0.01, ***p < 0.001

To account for these residual influences of diuresis on the concentrations of Cd and proteins in urine, we pursued the analyses by expressing the concentrations of urinary analytes per liter or per g of creatinine and by testing different models of adjustment for diuresis. Table 3 shows that the associations of urinary RBP and protein HC with U-Cd disappear or loose most of their statistical significance after adjustment for current smoking (protein HC) and for the residual influence of diuresis on the basis of urinary creatinine. Quite unexpectedly, the protein which consistently correlated with U-Cd whatever the model was urinary albumin. These results were unchanged when testing pack-years or current smoking alone or when removing age from the list of predictors.

**Table 3:** Predictors of RBP, protein HC and albumin in urine according to different modes of expression of results and adjustment for the residual influence of diuresis

	Concentrations of urinary markers per liter				Concentrations of urinary markers per g creatinine			
**Dependent variable**	**Independent variables**	**B**	**95% CI**	**P**	**Independent variables**	**B**	**95% CI**	**P**

RBP in urine	*Model 1*				*Model 1*			
	Log U-Cd	10.7	65.6 to 147.8	<0.001	Current smoking	27.4	9.1 to 46.0	0.004
					Log U-Cd	30.9	1.81 to 60.0	0.04
	*Model 2*				*Model 2*			
	Current smoking	34.2	10.3 to 58.2	0.005	Current smoking	27.4	9.1 to 46.0	0.004
	Log U-Cd	34.7	-3.83 to 73.3	0.08	Log U-Cd	30.9	1.81 to 60.0	0.04
	Log U-Cr	285.7	220.0 to 350.6	<0.001				
	*Model 3*				*Model 3*			
	Current smoking	34.2	10.3 to 58.2	0.005	Current smoking	28.8	10.3 to 47.3	0.003
	Log U-Cd adj	34.7	-3.82 to 73.3	0.07	Log U-Cd adj	25.4	-4.5 to 55.2	0.10
	Log U-Cr	308.0	248.0 to 367.3	<0.001				

Log U-Protein HC	*Model 1*				*Model 1*			
	Age	0.009	0.005 to 0.013	<0.001	Age	0.009	0.005 to 0.014	<0.001
	Current smoking	0.14	0.054 to 0.22	0.001	Current smoking	0.12	0.02 to 0.22	0.02
	Log U-Cd	-0.039	-0.16 to 0.086	0.54	Log U-Cd	0.13	-.033 to 0.29	0.12
	*Model 2*				*Model 2*			
	Age	0.007	0.003 to 0.011	<0.001	Age	0.008	0.003 to 0.13	0.002
	Current smoking	0.11	0.03 to 0.20	0.008	Current smoking	0.011	0.18 to 0.21	0.02
	Log U-Cd	0.05	-0.084 to 0.19	0.46	Log U-Cd	0.081	-0.079 to 0.24	0.32
	Log U-Cr	-0.36	-0.59 to -0.13	0.003	Log U-Cr	-0.47	-0.72 to -0.22	<0.001
	*Model 3*				*Model 3*			
	Age	0.007	0.003 to 0.011	<0.001	Age	0.008	0.003 to 0.013	0.002
	Current smoking	0.11	0.03 to 0.20	0.008	Current smoking	0.011	0.018 to 0.21	0.02
	Log U-Cd adj	0.05	-0.084 to 0.19	0.46	Log U-Cd adj	0.081	-0.079 to 0.24	0.32
	Log U-Cr	-0.33	-0.54 to -0.12	0.003	Log U-Cr	-0.52	-0.75 to -0.25	<0.001

Log U-ALB	*Model 1*				*Model 1*			
	Log U-Cd	0.39	0.201 to 0.58	<0.001	Log U-Cd	0.19	0.012 to 1.00	0.04
	*Model 2*				*Model 2*			
	Log U-Cd	0.20	0.02 to 0.39	0.03	Log U-Cd	0.19	0.012 to 1.00	0.04
	Log U-Cr	0.86	0.54 to 1.18	<0.001				
	*Model 3*				*Model 3*			
	Log U-Cd adj	0.20	0.02 to 0.39	0.03	Log U-Cd adj	0.20	0.016 to 0.39	0.03
	Log U-Cr	0.99	0.69 to 1.29	<0.001				

U-Cdadj, concentration of Cd expressed per g of creatinine and adjusted for the residual influence of urinary creartinine on the basis of the simple regression coefficient (Figure 1). The regression coefficients were estimated by stepwise multiple regression analyses testing as predictors age, BMI, Pb in blood, Zn in urine, current smoking, number of pack-years (>0-15 and >15).

To avoid confounding by diuresis, we repeated the above analyses by using B-Cd as indicator of Cd exposure. The only significant determinants entering in the different models (models 1 and 2) were current smoking for urinary RBP, number of pack-years >15 for albumin, and B-Cd with age for urinary protein HC. The association between urinary protein HC and B-Cd was, however, driven by tobacco smoking for it completely disappeared after exclusion of smokers. No association was found between lead in blood and urinary proteins. After stratification for smoking status, an association emerged between B-Cd and urinary protein HC in current smokers (p = 0.04) and between urinary RBP and B-Cd in never smokers (p = 0.02).

**Figure 2:**
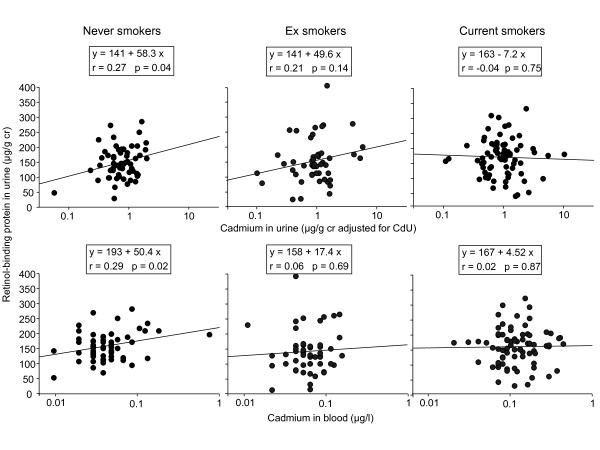
**Associations between urinary RBP and the concentrations of Cd in urine or blood in workers stratified in never, ever and current smokers**. Concentrations of Cd in urine, expressed per g of creatinine, have been further adjusted for the residual influence of diuresis on the basis of the simple regression coefficient between log U-Cd and log urinary creatinine.

The confounding effects of smoking in the assessment of the renal effects of Cd are illustrated in Figure 2 displaying the relationships of urinary RBP with U-Cd and B-Cd. To ensure a complete elimination of the influence of diuresis, the concentration of Cd in urine was expressed per g of creatinine and further adjusted for the residual effect of diuresis on the basis of the simple regression coefficient (Table 2, model 3). Figure 2 clearly shows that urinary RBP correlates significantly with U-Cd (p = 0.04) and even more with B-Cd (p = 0.02) only in never smokers. These associations did not emerge in ex smokers (p = 0.33 and 0.15) and even less in current smokers, (p = 0.74 and 0.87, respectively), which clearly means that the higher urinary excretion of RBP associated with smoking cannot be explained by their higher exposure to Cd. The strong correlations between RBP and Cd levels in urine and blood observed in the whole population (Table 2) appear thus to be largely driven by the increases of Cd exposure and of urinary RBP, which occur independently in chronic smokers.

**Figure 3:**
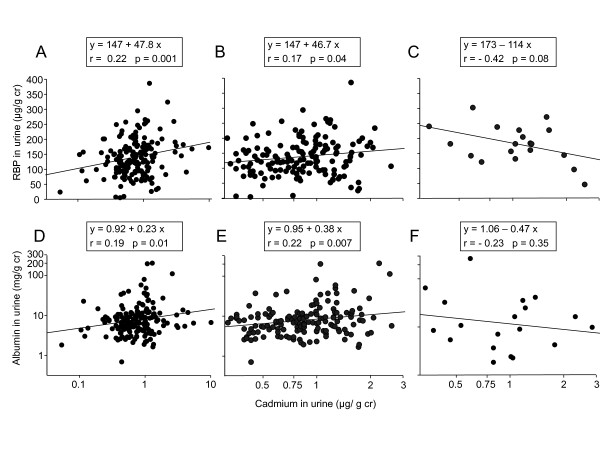
**Associations between RBP and Cd in the urine of workers according to the integrity of the glomerular filter (panels A and B, U-ALB<27 mg/g creatinine, panel C, U-ALB>27 mg/g cr mg/g creatinine) and between albumin and Cd according to the integrity of the tubular function (panels D and E, U-RBP<220 μg/g creatinine, panel F, U-RBP>220 μg/g creatinine)**. The panels B and E represent the same relationships as respectively the panels A and D but by limiting the U-Cd scale to that of the panels C and F in order to ensure that differences in these relationships were not due to differences in U-Cd levels. The cut-off values used to stratify the population correspond to the 90^th ^percentiles of values obtained over the whole population.

We further analyzed our data by examining to what extent associations between RBP and Cd in urine were influenced by the integrity of the glomerular or tubular function. As shown in Figure 3, the associations of RBP or albumin with Cd in urine completely disappeared in subjects with increased albuminuria (>90^th ^percentile or 27 mg/g cr) or increased urinary RBP (>90^th ^percentile or 220 μg/g cr), respectively (panels C and F in Figure 3). This phenomenon is not the consequence of differences in Cd exposure levels between subjects with a normal or an increased urinary excretion of albumin or RBP. Indeed, the associations between these two proteins and U-Cd persist in subjects with exactly the same range of U-Cd values (0.2-3 μg/g cr) as in subjects with increased albumin or RBP in urine (Figure 3, panel B and E).

**Figure 4:**
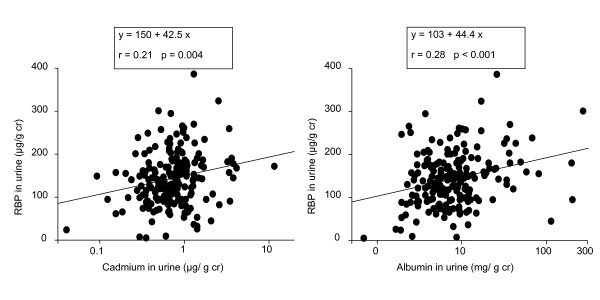
**Comparison of the associations of RBP with Cd and albumin in the urine of workers**.

We also tried to assess to what extent the associations of albumin and RBP with Cd in urine were independent of each other by mutually adjusting their concentrations in multiple regression analyses. The association between RBP and urinary Cd was no more statistically significant when considering albumin as a potential predictor. Likewise, the association between albumin and Cd in urine disappeared after adjustment for urinary RBP. This closed interdependence of Cd, RBP and albumin in urine also emerges from Figure 4 which shows that, plotted on the same scales, the relationship between RBP and Cd in urine is almost the replicate of that linking RBP to albumin (regression coefficient, 42.5 vs 44.4, respectively).

## Discussion

Our study confirms the existence of associations between urinary Cd and LMW proteins in urine in subjects with low exposure levels to Cd. These associations, however, were progressively abolished by the adjustment of LMW proteins for current smoking, age and the residual influence of diuresis. We found, however, that the urinary excretion of Cd was consistently associated with the excretion of albumin through a relationship, which was unaffected by diuresis, age or smoking. Stratification of workers according to the level of urinary albumin or RBP revealed that these proteins were mutually influencing their association with urinary Cd. When using blood Cd as exposure indicator, a significant association emerged only with urinary RBP but again this association was largely driven by current smoking. Of note also, in models based on B-Cd, urinary albumin was associated with cumulative smoking >15 pack-years.

The increased excretion of LMW proteins is most probably due to the renal toxicity of tobacco smoke. Several studies have indeed shown that chronic smoking, even moderate, is associated with an increased urinary excretion of proteins [16-19]. The proteinuria described in these studies appears to be primarily glomerular with a marked increase in the urinary excretion of albumin. Although in the model based on B-Cd, urinary albumin was increased in workers with the highest pack-year smoking history, the albumin excretion of current smokers was on average not different from that of never smokers. A possible explanation for this lack of increase in albumin excretion might be that our population included relatively few heavy smokers. This is probably due to the relatively young age of the study population along perhaps also, especially in smelters, a healthy worker effect. Our data suggest thus that smoking can cause an impairment of tubular function which might be reversible as it was found only in current but not in ex-smokers. The component of tobacco smoke impairing tubular function is unknown but it does not seem that Cd could be the causative agent. One reason is that the effect of smoking was found in models adjusting the LMW protein excretion for the U-Cd level. The other reason is that LMW proteinuria was associated only with current smoking and showed no relationship with number of pack-years, which would have been expected in case of a chronic effect due to the accumulation of Cd.

Among the determinants of Cd and protein concentrations in urine, the residual influence of diuresis after adjustment for urinary creatinine appears as the most confusing one, especially as this factor did not affect urinary analytes in the same way. The urinary excretion of RBP and albumin expressed per g of creatinine was no more correlated with urinary creatinine, meaning that the adjustment for urinary creatinine completely eliminates the influence of diuresis. By contrast, the concentrations of urinary Cd expressed per g of creatinine displayed a strong inverse association with urinary creatinine. Dividing urinary Cd per g of creatinine results thus in a sort of over-adjustment, which necessitates a correction based on the regression coefficient to completely eliminate variations linked to diuresis. The situation is even more confusing with protein HC as the adjustment for urinary creatinine amplified an association with urinary creatinine, which paradoxically was already negative with protein HC concentrations expressed per liter. The reasons for these inconsistent and paradoxical associations of Cd and proteins with urinary creatinine are unknown. But clearly, the lack of consideration for these residual influences of diuresis might generate artifactual associations between Cd and proteins arising from inaccuracies in the adjustment for urine dilution.

The finding that associations of RBP and albumin with U-Cd are abolished in subjects with respectively glomerular and tubular impairment suggests that these associations are generated by distinct co-excretion mechanisms. The first mechanism would be an increased excretion to Cd bound to proteins reabsorbed by the proximal tubule, in particular to metallothionein, which has been shown to follow the same glomerular filtration-tubular reabsorption pathway as other LMW proteins [20]. The association between urinary RBP and U-Cd found in subjects with normal values might thus simply reflect of the physiological variation in the tubular reabsorption capacity of the proximal tubule. The second co-excretion mechanism would be an enhanced excretion of Cd accompanying the albumin excretion. One possibility would be that Cd is excreted in greater amounts because of its binding to albumin, the main B-Cdinding protein in plasma [21]. Another possibility which is suggested by the similarity of the relationships of RBP with Cd and albumin in urine, would be a competitive inhibition by albumin of the tubular reabsorption of LMW proteins, including Cd-metallothionein. These different mechanisms are supported by the experimental evidence that albumin can competitively inhibit the tubular uptake of LMW proteins such as RBP and β_2_-microglobulin [22,23]. They are also in accordance with our current understanding of the tubular reabsorption of proteins. It is now well established that proteins are removed from the tubular fluid by receptor-mediator endocytosis via megalin, a receptor known to bind a large variety of proteins, including albumin, metallothionein and the LMW proteins used for screening tubular proteinuria [24,25].

In conclusion, our study shows that associations of proteinuria with low-level urinary Cd are largely driven by current smoking, variations in diuresis and probably also the co-excretion of Cd with proteins. The mechanism underlying the association between smoking and the excretion of LMW proteins remains to be elucidated but our data do not support an implication of Cd.

## Abbreviations

B-Cd: cadmium in blood; U-Cd: cadmium in urine; B-Pb: lead in blood; U-Zn: zinc in urine; U-RBP: retinol-binding protein in urine; U-Protein HC: protein HC in urine; U-ALB: albumin in urine; IQR: interquartile range; BMI: body mass index

## Competing interests

The authors declare that they have no competing interests.

## Authors' contributions

NH was responsible for the study design and participated to the samples and data collection kidney biomarkers measurement, data analysis and manuscript writing. SS was responsible for the collection of biological samples of workers. XD was responsible for the kidney markers analyses. VH was responsible for metal analyses and DL contributed to the design of the study. AB was responsible for data analysis, results interpretation and manuscript writing. All authors read and approved the final manuscript.
